# Fibroblast growth factor signaling in macrophage polarization: impact on health and diseases

**DOI:** 10.3389/fimmu.2024.1390453

**Published:** 2024-06-19

**Authors:** Luyao Shen, Yongsheng Li, Huakan Zhao

**Affiliations:** ^1^ The Second Affiliated Hospital & Yuying Children’s Hospital/The Second School of Medicine, Wenzhou Medical University, Wenzhou, China; ^2^ Department of Medical Oncology, Chongqing University Cancer Hospital, Chongqing, China

**Keywords:** fibroblast growth factor, fibroblast growth factor receptor, macrophage polarization, disease, signaling pathway

## Abstract

Fibroblast growth factors (FGFs) are a versatile family of peptide growth factors that are involved in various biological functions, including cell growth and differentiation, embryonic development, angiogenesis, and metabolism. Abnormal FGF/FGF receptor (FGFR) signaling has been implicated in the pathogenesis of multiple diseases such as cancer, metabolic diseases, and inflammatory diseases. It is worth noting that macrophage polarization, which involves distinct functional phenotypes, plays a crucial role in tissue repair, homeostasis maintenance, and immune responses. Recent evidence suggests that FGF/FGFR signaling closely participates in the polarization of macrophages, indicating that they could be potential targets for therapeutic manipulation of diseases associated with dysfunctional macrophages. In this article, we provide an overview of the structure, function, and downstream regulatory pathways of FGFs, as well as crosstalk between FGF signaling and macrophage polarization. Additionally, we summarize the potential application of harnessing FGF signaling to modulate macrophage polarization.

## 1 Introduction

Fibroblast growth factors (FGFs) are polypeptides composed of approximately 150–200 amino acids. There are 18 endocrine molecules and 4 intracellular FGF homologs (1). Unlike vascular endothelial growth factor (VEGF), which is specific to endothelial cells, FGF acts on various types of cells by binding to their corresponding FGF receptors (FGFR). Most FGFs have a strong affinity for heparin and are associated with extracellular matrix (ECM) components (2). After binding with FGFR monomers, FGF induces a molecular conformational change in FGFR and activates the tyrosine kinase (TK) by phosphorylating tyrosine residues on the cytoplasmic side of FGFR (3). These phosphorylated tyrosine residues serve as docking sites for downstream molecules, initiating a sequence of signaling cascades (4). One of these pathways is the canonical FGF/FGFR signaling pathway, which involves Ras/Raf-MEK-MAPKs (mitogen-activated protein kinases), phosphatidylinositol-3 kinase/protein kinase (PI3K/AKT), phospholipase C gamma (PLCγ), and signal transducer and activator of transcription (STAT). By modulating these signaling pathways, FGF/FGFR signaling plays a crucial role in orchestrating various cellular functions, including cell proliferation, apoptosis, survival, metabolism, morphogenesis, and differentiation.

FGF/FGFR signaling also plays a crucial role in regulating various biological functions, such as embryonic development, angiogenesis, tissue homeostasis, wound repair, and cancer development (1, 5). For example, FGF/FGFRs are involved in promoting human skeletal development, maintaining homeostasis, and aiding in the repair of bone and cartilage after injury (6). During lung development, as well as the development of cardiac, vascular, and lymphatic vessels, there is widespread expression of FGFs and their ligands (7, 8). Abnormalities in the metabolism of the FGF/FGFR signaling axis have been extensively studied in various diseases, including congenital cranial suture atresia, dwarf syndrome, chronic kidney disease (CKD), obesity, insulin resistance, and different types of tumors (9).

In recent years, there has been a growing interest in the role of the FGF signaling pathway in the immune system. Macrophages, a type of immune cell, play a crucial role in various aspects of the organism, including development, homeostasis, and tissue repair (10). They are also involved in the immune response against pathogens. However, continuous damage can disrupt the normal functioning of macrophages, leading to diseases such as fibrosis, obesity, inflammation, and tumors. Macrophages have the ability to adapt and respond to external changes, enabling them to recognize and react to alterations in tissue physiology and the environment. The functional classification of macrophages can be divided into two categories based on their response to inflammatory states: classically activated macrophages (M1) and alternatively activated macrophages (M2) (11). These two types of macrophages are induced to polarize by different signaling molecules. Moreover, as an essential component of the tumor microenvironment, macrophages have been found to be extensively regulated by FGF/FGFRs (12–14). Therefore, gaining a deeper understanding of the function and mechanism of FGF signaling in macrophage polarization will be valuable for developing new therapeutic strategies for multiple diseases associated with dysregulated macrophages.

In this paper, we present a thorough examination of the members of the FGF family and their structures, as well as their downstream pathways. We also delve into the regulatory role of the FGF/FGFR signaling pathway and its influence on macrophage polarization. Furthermore, we provide a summary of the current research on the regulatory effects of FGF family members on diseases that are regulated by macrophage polarization.

## 2 FGF signaling axis

**Figure 1:**
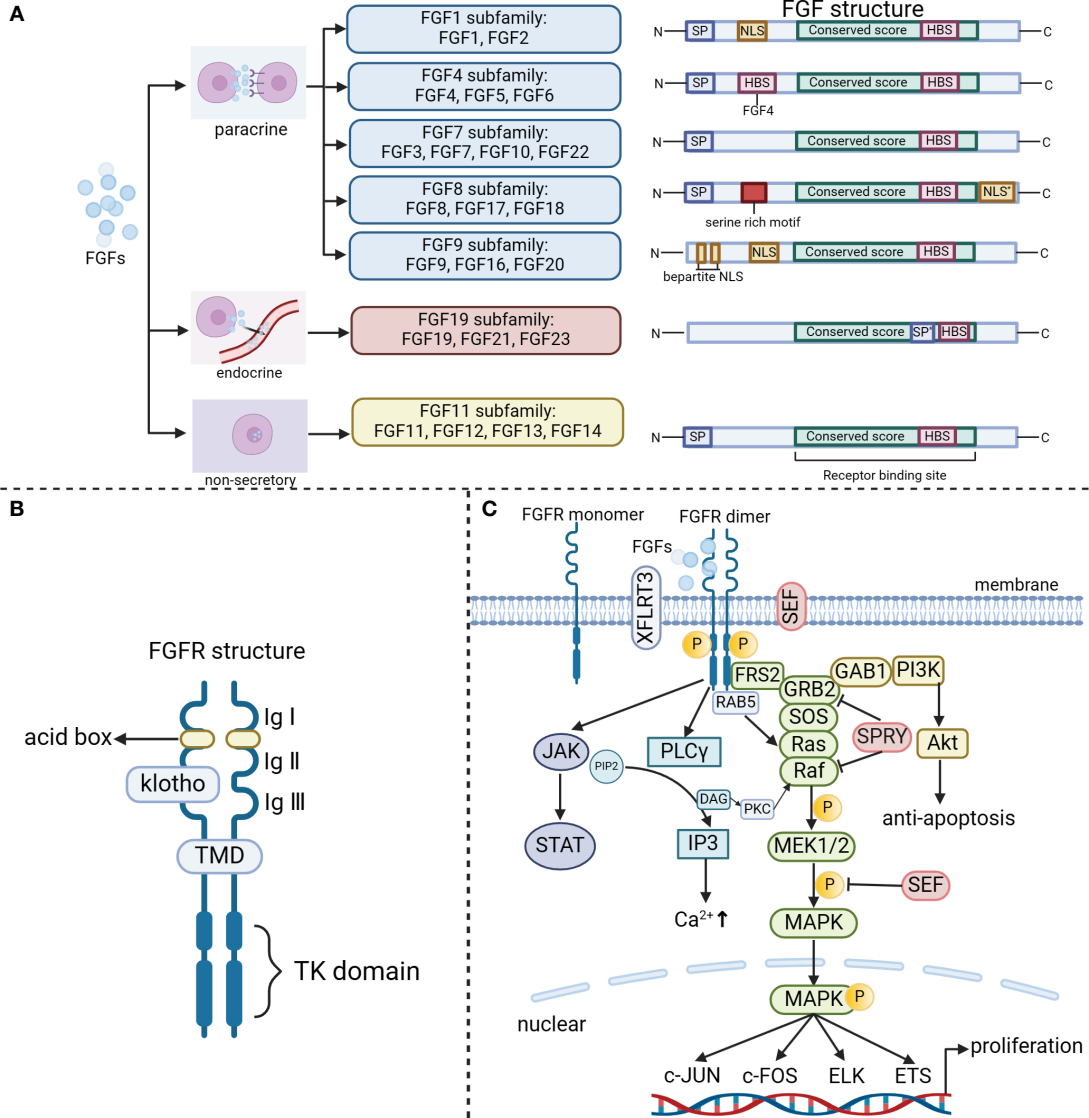
The classical FGF/FGFR pathways. **(A)** The FGF family includes the paracrine subfamilies (FGF1, FGF4, FGF7, FGF8, FGF9 subfamily), the endocrine FGF19 subfamily, and the non-secretory FGF11 subfamily. Based on their secretion/action mechanism, the family members have been classified. N (amino terminus); SP (signal peptide/propeptide); NLS (nuclear localization signal); NLS*(nuclear localization signal at C-terminu); HBS (heparin binding site); SP*[uncleaved bipartite signal sequence (secreted)]; C (carboxy terminus, serine-rich motif); Bipartite NLS. **(B)** FGFR consists of three extracellular immunoglobulin (Ig) structural domains, two acid boxes, a transmembrane helical structural domain (TMD) and an intracellular tyrosine kinase structural domain. **(C)** The binding of appropriate growth factors to receptors triggers conformational changes in FGFRs, leading to their dimerization and activation. Once activated, FGFRs phosphorylate FRS2, which then binds to the SH2 domain-containing adaptor GRB2. Subsequently, GRB2 binds to SOS, GAB1, and activates Ras/Raf/MAPKs, including ERK MAPK, p38 MAPK, and JNK MAPK. In addition, the activated FGFRs also activate phosphatidylinositol (PI)-3 kinase and STAT. Furthermore, FGFRs recruit and phosphorylate PLCγ. Within the FGF synexpression group, SEF and XFLRT3 are transmembrane proteins that can directly interact with FGFRs. SEF acts as a negative regulator by influencing the phosphorylation of the MAPK ERK cascade. On the other hand, XFLRT3 forms a complex with FGF receptors and enhances FGF/FGFR signaling. RAB5, a small GTPase, plays a role in maintaining the RAS-MAPK signaling pathway. Lastly, Spry functions to attenuate FGF/FGFR signaling either at the level of GRB2 or at the level of Raf.

In the 1930s, scientists made a significant discovery of a peptide called FGF in the secretions of the pituitary and hypothalamus. FGF has the remarkable ability to stimulate the proliferation of fibroblasts. So far, researchers have identified 28 different members of the FGF family, making it the most diverse growth factor found in vertebrates. Among mice and humans, 22 FGF ligands have been confirmed, with molecular weights ranging from 17–34 kDa. These members share amino acid homology ranging from 13% to 71% and have highly conserved gene structures and amino acid sequences. Based on sequence homology and phylogeny (15), these 22 FGFs can be classified into six subfamilies, which include five paracrine subfamilies, one endocrine subfamily, and a non-secretory FGF11 subfamily. The paracrine FGF families consist of the FGF1 subfamily (FGF1, FGF2), FGF4 subfamily (FGF4, FGF5, FGF6), FGF7 subfamily (FGF3, FGF7, FGF10, FGF22), FGF8 subfamily (FGF8, FGF17, FGF18), and FGF9 subfamily (FGF9, FGF16, FGF20). The FGF19 subfamily (FGF19, 21, and 23) is unique among other FGF subfamily members as it emits signals in an endocrine manner dependent on the presence of klotho proteins in their target tissues (16) (**Figure 1A**). FGF15 serves as the mouse equivalent of human FGF19 (17). Additionally, non-secretory FGF11 subfamily (FGF11, FGF12, FGF13, FGF14) are localized in the nucleus, and do not activate the FGF receptors.

### 2.1 Components of FGF signaling

Paracrine FGFs and endocrine FGFs have distinct structural characteristics. Paracrine FGFs possess a conserved β-trefoil fold consisting of 12-stranded β-sheets (β1–β12), which is a result of a shared FGF core homology domain (∼125 amino acids). On the other hand, endocrine FGFs exhibit an atypical trefoil fold that lacks the β11 strand (18). The FGF core trefoil domain is surrounded by highly variable amino terminal and carboxy terminal regions, which differ in length and amino acid sequence among different FGFs (**Figure 1A**). These variations in the terminal regions contribute to the diverse biological functions of different FGFs by influencing their binding to receptors and co-receptors (19).

The mammalian FGF family interacts with four TK receptors (FGFR1, FGFR2, FGFR3, and FGFR4) (20), through which it exerts its functions. These receptors are highly conserved and have a crucial role in triggering intracellular signaling cascades that mediate the bioactivity-related responses of FGF. Additionally, there is an additional receptor called65 FGFR5 (also known as FGFRL1) that binds FGFs (21). Unlike other receptors, FGFR5 does not have a TK structural domain and is believed to potentially regulate signaling in a negative manner. FGFRs are transmembrane proteins with three main structural domains (3, 18, 22): an extracellular domain, a transmembrane domain (TMD), and an intracellular TK domain (**Figure 1B**). The extracellular domain of the receptor consists of three immunoglobulin (Ig)-like domains, along with two acid boxes. It also includes the heparin-binding motif of FGF, the heparin cofactor, and the chaperone protein. The TMD anchors the receptor to the cell membrane and facilitates its dimerization. Within the cytoplasm, the proximal membrane region of FGFR is responsible for receptor dimerization, while the intracellular kinase structural domain is essential for FGF-related signaling (22).

To activate one of the four cell surface FGFRs, paracrine FGFs bind with high affinity to heparan sulfate proteoglycan (HSPG) and require the presence of acetyl heparan sulfate in a synergistic manner (23). Typically, HSPG binds with FGFs at the β1–β2 loop and the extended β10–β12 region of FGFs. However, members of the FGF19 subfamily lack the paracrine-conserved glycine box and the truncated β10–β12 region, resulting in negligible binding affinity between HSPG and the FGF19 subfamily members (24). This unique feature allows these FGFs to permeate through the HSPG-abundant ECM and enter the bloodstream, where they exert their regulatory roles similar to endocrine hormones. In addition, endocrine FGFs also exhibit poor affinity for their cognate FGFRs, resulting in ineffective endocrine FGF/FGFR binding and dimerization. The Klotho proteins, α/β Klotho, are essential for the high-affinity binding of endocrine FGFs to their cognate FGFRs (25). The Klotho coreceptors efficiently bind to the c-splice isoforms of FGFR1–3 and FGFR4 to form the FGFR-Klotho complex, promoting their binding with FGFs and dimerization, thereby reinforcing endocrine FGF/FGFR signaling specificity. In summary, successful endocrine FGF/FGFR signaling relies on the interaction with FGFRs and Klothos.

### 2.2 FGF downstream pathways

FGFRs can be activated by FGF ligand-dependent dimerization, which induces conformational changes in the receptor structure (26, 27). This activation triggers the activation of the intracellular kinase structural domain and enables intermolecular transphosphorylation of the TK structural domain and intracellular region. The phosphorylated tyrosine residues on the receptor act as docking sites for the junction protein, which can be directly phosphorylated by FGFR. This phosphorylation event leads to the activation of multiple downstream signaling pathways (**Figure 1C**).

FGFR substrate 2 (FRS2) is a crucial junction protein that primarily targets FGFR. It gets phosphorylated by the activated FGFR at multiple sites (27). FRS2 interacts with the proximal membrane region of FGFR through its phosphotyrosine binding (PTB) structural domain. This interaction facilitates the recruitment of son of sevenless (SOS protein) and growth factor receptor binding protein 2 (GRB2), ultimately leading to the activation of the Ras/Raf/MAPK pathway. Additionally, GRB2-associated binding protein 1 (GAB1) recruits PI3K, which activates the AKT-dependent anti-apoptotic pathway (28). Furthermore, activated FGFRs also activate PI3K and STAT. In summary, FGF signaling is transduced to the RAS-MAPK or PI3K-AKT signaling cascades through FRS2 and GRB2 (28).

Phosphorylation of tyrosine residues in the c-terminal region of the FGFR facilitates the recruitment of PLC-γ binding sites, leading to the activation of PLC-γ. This activation catalyzes the conversion of phosphatidylinositol diphosphate (PIP2) into diacylglycerol (DAG) and inositol triphosphate (IP3) (29). Subsequently, the release of Ca^2+^ from the endoplasmic reticulum (ER) mediated by PLCγ-IP3 triggers stromal interaction molecule 1 (STIM1)-regulated influx of Ca^2+^ through store-operated calcium entry (SOCE). This influx promotes the activation of Ca^2+^-dependent signaling pathways, such as calcium/calmodulin-dependent protein kinase II (CaMKII) and calcineurin (CaN) (30).

Within the FGF co-expression group, SEF and XFLRT3 are transmembrane proteins that directly interact with FGFR. SEF negatively regulates the MAPK ERK cascade by influencing its phosphorylation (31). On the other hand, XFLRT3 forms a complex with the FGF receptor and enhances FGF/FGFR signaling (31). In contrast, SPRY acts on GRB2 and/or RAF to dampen FGF/FGFR signaling (32). Moreover, FGF19/15 activates FGFR4 to recruit and phosphorylate neurofibromatosis type 2 (NF2), which relieves the inhibitory effect of Raf on the mammalian sterile 20-like kinase (Mst1/2), thereby stimulating hippo signaling to suppress bile acid metabolism (33). Furthermore, FGF signaling can also activate SRC TKs. The activation of RAS-MAPK by FGF is associated with cell proliferation, while the activation of PI3K-AKT is involved in cell survival (34). RAB5, a small GTPase, acts as a binding partner of activated FGFR and plays a role in maintaining the RAS-MAPK signaling pathway, but not the PI3K-AKT signaling pathway (35).

Intracellular FGFs are unable to be secreted and do not have any known interaction with FGFRs (36). However, several proteins have been identified to directly interact with intracellular FGFs. These proteins include voltage gated sodium channels (VGSC), MAPK8-interacting protein 2 (MAPK8IP2), β-tubulin, and NEMO (NF-κB essential modulator) (37–40). For example, intracellular FGFs regulate the subcellular localization of Nav channels at the axon initial segment during development by interacting with the cytosolic carboxy terminal tail of VGSC (37). FGF12 has been shown to interact with IB2, promoting ERK protein phosphorylation but not affecting AKT phosphorylation (40). Overall, FGF signaling activates multiple downstream signaling cascades, resulting in unique and extensive physiological regulatory functions.

### 2.3 Functions of FGF signaling

Aside from their pro-divisive effects on fibroblasts, FGF signaling plays a crucial role in a variety of biological processes. These processes include development, tissue repair, metabolism, tissue homeostasis, and cancer development. FGF signaling achieves these effects through autocrine, endocrine, and paracrine pathways.

#### 2.3.1 Effects on development

Several studies have implicated FGF signaling in embryonic development, showing that FGFs have a unique spatiotemporal expression pattern at each stage (41). FGFs are involved in various processes such as cell migration during gastrulation, epithelial-mesenchymal transition (EMT) during limb morphogenesis, neural induction, and patterning in later stages of development (41). Mutations in FGFRs or FGFs, whether resulting in increased activity (gain-of-function mutations) or reduced activity (loss-of-function mutations), can cause inherited skeletal disorders in humans (42). For instance, excessive expression of FGF3 and FGF4 genes has been associated with cranial suture atresia. FGF1 has been found to play a crucial role in regulating the differentiation of bone marrow stromal cells (BMSCs) by inhibiting osteogenesis and promoting adipogenesis (43). Additionally, overexpression of FGF2 leads to a decrease in bone mass and mineralization defects, negatively impacting bone formation (44).

#### 2.3.2 Effects on tissue repair

FGF/FGFR signaling play a crucial role in tissue repair, a complex physiological process involving various cell types such as fibroblasts, epithelial cells, smooth muscle cells, endothelial cells, and macrophages (45–47). For example, in a bleomycin model of lung injury and pulmonary fibrosis, FGF2 is expressed in lung epithelial and inflammatory cells. Fgf2(-/-) mice exhibited significantly increased mortality and weight loss in response to bleomycin, highlighting the essential role of FGF2 in epithelial recovery (48). Additionally, treatment with FGF21 has been shown to promote functional recovery from spinal cord injury by suppressing injury-induced autophagy (49). In a mouse model, knocking down Klotho noticeably delays cutaneous wound healing and increases the levels of proinflammatory cytokines (50).

#### 2.3.3 Effects on metabolism

Several members of the FGFs, particularly the FGF19 subfamily, have been identified as significant regulators of energy metabolism in both human and mouse. FGF15/19 has emerged as a key regulator that controls the homeostasis of bile acids (BA) and glucose (5). FGF15/19 is expressed in the ileal enterocytes under the transcriptional control of the BA-activated farnesoid X receptor (FXR), and it inhibits hepatic BA synthesis by suppressing the transcription of Cyp7A1, which is the rate-limiting enzyme (51, 52). Additionally, FGF15/19 enhances protein and glycogen synthesis while suppressing gluconeogenesis in the liver, without stimulating lipogenesis (53). Transgenic mice with FGF19 overexpression also exhibit increased energy consumption, enhanced fatty acid oxidation, and reduced fat synthesis (54). Decreased levels of FGF19 are commonly observed in obesity-related conditions such as type 2 diabetes (T2D), gestational diabetes, non-alcoholic fatty liver disease (NAFLD), and NASH, as well as in bile malabsorption conditions like cystic fibrosis (52).

FGF21 is primarily secreted by the liver and plays a crucial role in energy metabolism through various mechanisms. Its effects include promoting fatty acid oxidation, enhancing insulin sensitivity in tissue cells, and facilitating gluconeogenesis as well as ketone body synthesis (55, 56). Additionally, increasing levels of myogenic FGF21 can enhance skeletal muscle glucose uptake, fatty acid oxidation, and insulin sensitivity, resulting in improved lipid metabolism and weight loss (57). In mouse models of non-alcoholic fatty liver disease (NAFLD) and NASH, FGF21 and its analogs have been shown to act as hepatoprotective factors by directly regulating hepatic lipid and free fatty acid metabolism. This leads to effective inhibition of hepatic steatosis, reduction in fat production, and increased fat oxidation (58).

Differing from FGF15/19 and FGF21, FGF23 interacts with FGFR through α-klotho, which is mainly expressed in the kidney. The FGF23-αKlotho pathway regulates phosphate excretion in the kidney and decreases the synthesis of vitamin D and parathyroid hormone (PTH) (59). FGF23 is highly expressed in osteoblasts and osteoclasts and plays a crucial role in active bone remodeling in response to increased osteotriol, elevated phosphate and calcium levels, enhanced parathyroid hormone, iron and magnesium loss, and mechanisms dependent on the vitamin D receptor (5). Elevated serum FGF23 levels in patients with CKD lead to decreased osteotriol, resulting in secondary hyperparathyroidism (60). In animal models of CKD, FGF23 has also been observed to mediate cardiac calcium regulation and contractile function. Furthermore, chronically elevated levels of FGF23 can directly cause left ventricular hypertrophy and increased cardiovascular mortality in patients with CKD (59).

#### 2.3.4 Effects on tissue homeostasis

The relevance of FGFs/FGFRs to the inflammatory response has been extensively studied in recent years (61, 62). It has consistently been observed that FGF1, which is highly expressed in arthritic bone, cartilage, synovium, ligaments, and tendons, tends to exacerbate the inflammatory response (63). However, FGF21 is believed to have a role in reducing inflammation in the heart, liver, and kidney (64–66). In prostate cancer cells, FGFR1 promotes inflammation by activating the NF-κB signaling pathway (67). Additionally, the absence of FGFR3 in mice worsens joint damage by enhancing macrophage chemotaxis through the activation of the NF-κB/chemokine receptor 7 (CXCR7) signaling pathway (68).

#### 2.3.5 Effects on cancer

The deregulation of the FGF/FGFR signaling network has been implicated in the development and progression of various types of cancers, such as uroepithelial carcinoma, multiple myeloma, prostate cancer, and hepatocellular carcinoma (HCC) (9). The expression and mutation of FGF signaling molecules in tumors are closely linked to these processes. For example, FGF9 is highly expressed in the majority of non-small cell lung carcinoma (NSCLC) tumors, and its high expression is associated with a poor prognosis for NSCLC patients (69). Similarly, aberrant FGF19 and its receptor FGFR4 have been identified as oncogenic drivers for a subset of HCC, and their presence is associated with a poor prognosis for HCC patients (70, 71).

**Figure 2:**
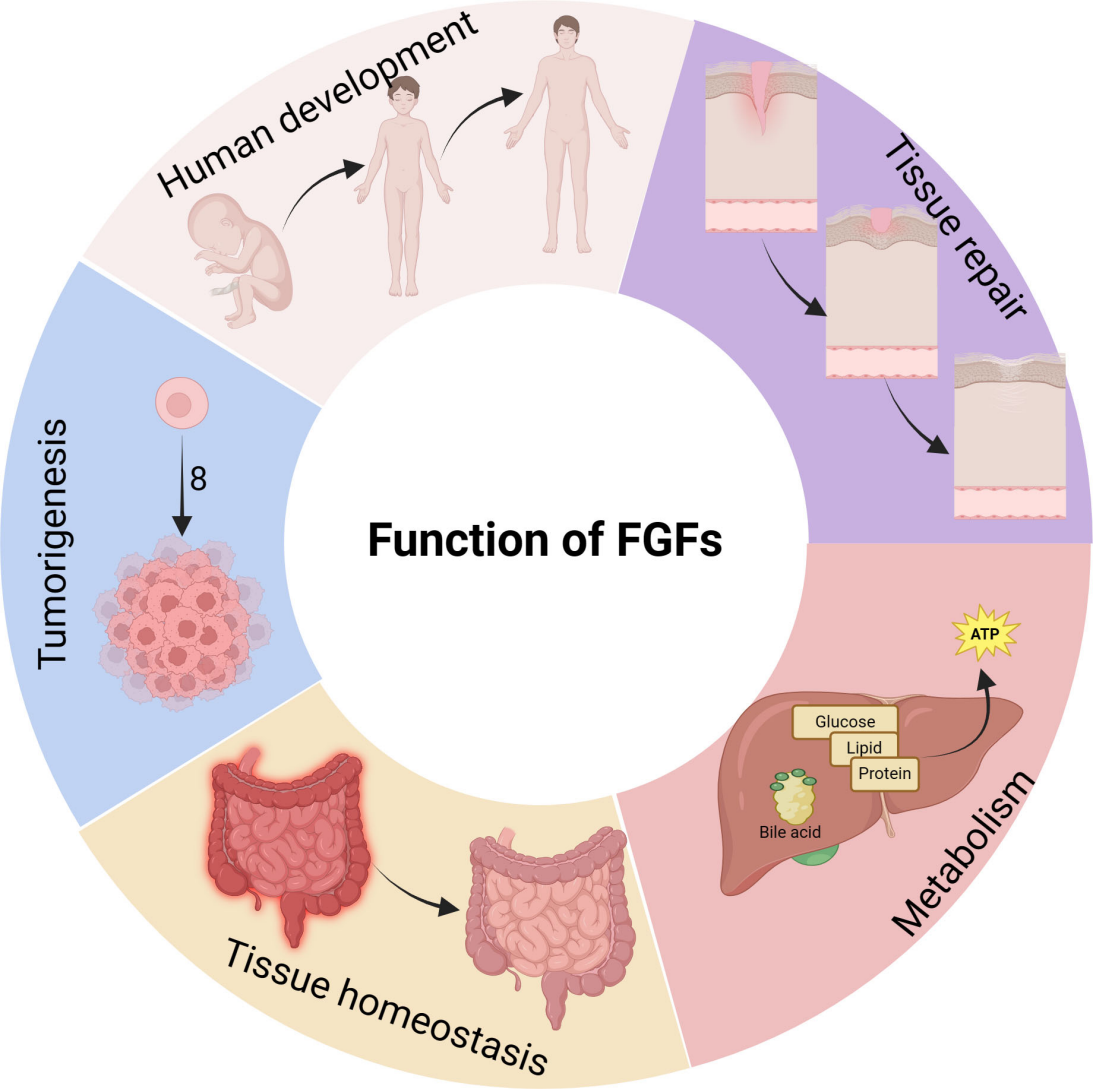
Biological functions of FGFs. FGFs have diverse regulatory functions in various physiological processes of the body. They not only promote the growth and development of bones and organs, but also contribute to the development of related diseases. FGFs are involved in tumorigenesis and metastasis, participate in inflammatory processes, and play a crucial role in nutrient metabolism.

The FGF/FGFR signaling pathway plays a significant role in various biological processes such as development, tissue repair, metabolism, tissue homeostasis, and cancer development, among others (**Figure 2**). It is important to note that these biological processes are closely associated with macrophages.

## 3 Macrophages: gatekeepers of homeostasis

Macrophages, as a fundamental component of the innate immune system, exhibit a remarkable capacity to adjust and specialize in reaction to diverse stimuli. They are crucial for upholding equilibrium within the body and safeguarding against invading pathogens. Circulating throughout the body, macrophages and monocytes are pivotal in various organs and tissues, each with its distinct nomenclature (72). Notably, microglia are situated in the central nervous system (CNS), Kupffer cells inhabit the liver, osteoclasts are found in bone, and alveolar macrophages populate the lungs (73). Specific populations of macrophages are also present in secondary lymphoid organs, such as marginal zone macrophages in the spleen, which regulate both innate and adaptive immunity towards apoptotic cells. Likewise, subenvelope sinus macrophages in the lymph nodes are responsible for eliminating viruses from the lymph and initiating antiviral immune responses. Within specific tissues, macrophages fulfill a range of functions, including phagocytosis of dead cells, debris, foreign antigens, and substances. Moreover, they orchestrate the inflammatory response and recruit additional macrophages as needed (74).

**Figure 3:**
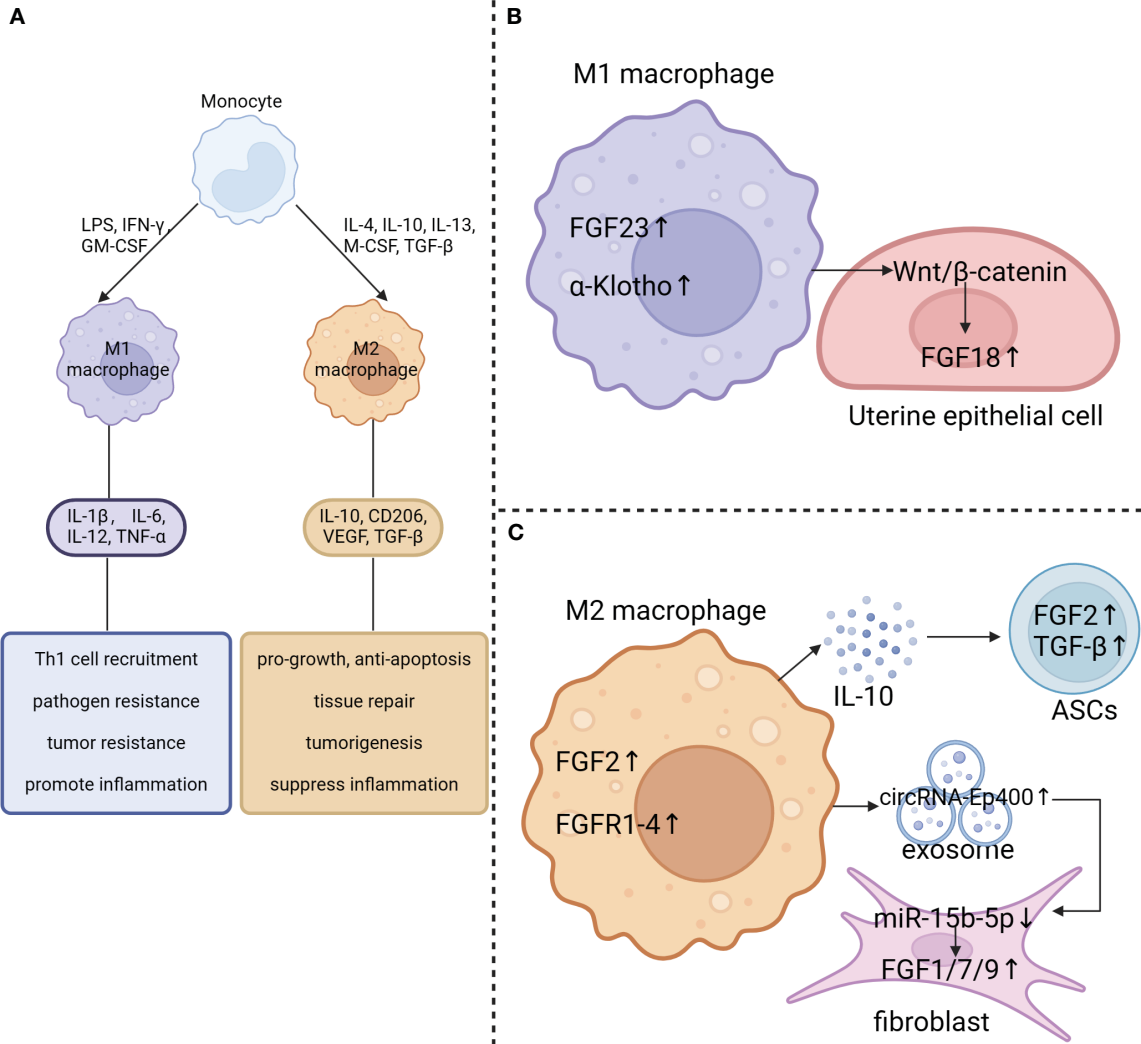
Effect of macrophage polarization on FGF signaling. **(A)** Blood-derived monocytes can be stimulated by GM-CSF, LPS, and IFN-γ to polarize into M1-type macrophages, which secrete the pro-inflammatory cytokines IL-1β, IL-6, IL-12, and TNF-α. On the other hand, M2 macrophages can be polarized by IL-4, IL-10, IL-13, M-CSF and TGF-β. They produce cytokines including IL-10, CD206, VEGF and TGF-β. **(B)** FGF23 and klotho are abundantly expressed in M1 macrophages. M1 macrophages also activate the Wnt/β-catenin pathway in uterine epithelial cells which results in upregulation of FGF18 expression. **(C)** FGF2 and FGFR1–4 are significantly elevated in M2 macrophage. Additionally, M2 macrophages may contribute to the upregulation of FGF2 and TGF-β expression in adipose-derived stem cells (ASCs) by secreting IL-10. Besides, exposomes derived from M2 macrophages with elevated circRNA-Ep400 suppresses miR-15b-5p activity in fibroblast, resulting in upregulation of FGF1/7/9 expression in fibroblasts.

Research has shown that macrophage phenotypes are influenced by the local microenvironment, allowing them to respond effectively to pathogens and signaling molecules (75, 76). This flexibility, known as polarization, results in a diverse range of macrophage functions (76). Macrophages are typically categorized into three groups: naive macrophages (Mφ or M0), which can differentiate into pro-inflammatory (M1) and anti-inflammatory (M2) phenotypes (**Figure 3A**). M1 macrophages, or classically activated macrophages, are involved in inflammation, pathogen clearance, and antitumor activities, characterized by strong antigen-presenting abilities and high expression of pro-inflammatory cytokines, such as interleukin (IL)-1β, IL-6, IL-12, and tumor necrosis factor-alpha (TNF-α) (75, 77). On the other hand, M2 macrophages, or alternatively activated macrophages, exhibit anti-inflammatory and immunomodulatory effects (78).

The balance between M1 and M2 macrophages is crucial in determining disease outcomes. Excessive M1 activation can lead to inflammatory diseases, while uncontrolled M2 activation is linked to immunosuppression-related diseases. The polarization of M1/M2 macrophages determines the fate of organs during severe infection or inflammation. Unrestrained M1 macrophages are associated with chronic inflammatory diseases like atherosclerosis, asthma, inflammatory bowel disease (IBD), rheumatoid arthritis (RA), and liver fibrosis (79). On the other hand, M2 macrophages contribute to reducing inflammation, promoting tissue repair, remodeling, angiogenesis, and maintaining homeostasis by secreting high levels of IL-10 and transforming growth factor beta (TGF-β) (79). Chronic inflammation is recognized as a key factor in cancer development, with M2-like TAMs playing a critical role in promoting tumor progression, growth, invasion, metastasis, and drug resistance (80). High infiltration of the M2-subtype is correlated with poor patient outcomes.

## 4 Crosstalk between FGFs and macrophages

Macrophages are being recognized for their crucial role in regulating disease and physical health. Recent studies have provided substantial evidence indicating a close relationship between macrophage function and polarization state with FGF signaling. It has been observed that the expression of FGF/FGFRs is modified during the process of macrophage polarization and phenotypic transition. Additionally, FGF signaling plays a significant role in regulating the polarization and function of macrophages.

### 4.1 FGF/FGFRs expression during macrophage polarization

The regulation of FGF signaling is tightly controlled under normal physiological conditions (1). It has been observed that macrophages in different polarization states play a crucial role in this regulation (**Figures 3B, C**). FGF23, a hormone produced by the bones, functions by activating the FGFR/αKlotho complex in the renal tubules, which helps to control the reabsorption of phosphate and the metabolism of vitamin D. Normally, macrophages do not express FGF23, but there is a significant increase in FGF23 expression in M1 macrophages induced by LPS/IFN-γ stimulation (13). Additionally, the expression of FGF2 in macrophages is considerably higher than in MDSCs, granulocytes, CD4^+^ T cells, and CD8^+^ T cells. Furthermore, TAMs isolated from MC38 tumors, which exhibit an M2-like phenotype, express several hundred times more Fgf2 RNA than BMDMs (14). Moreover, TAMs also show higher mRNA levels of Fgfr1, 3, and 4, with a particularly significant 100-fold increase in Fgfr2 compared to BMDMs (14). This indicates that tumor cells can influence macrophages to induce the expression of both FGFs and FGFRs. In addition, the treatment of THP-1-derived macrophages with Indoxyl sulfate (IS) promotes M1 polarization, leading to increased production of pro-inflammatory cytokines such as TNF-α, IL-6, and IL-1β, while also downregulating Klotho expression (14).

In addition, the polarization of macrophages can influence the expression of FGF components in nearby cells. M1 macrophages have been found to enhance the uterine Wnt/β-Catenin signaling pathway, leading to a significant increase in downstream FGF18 mRNA levels (81). This upregulated FGF18 acts on the uterine epithelium in a paracrine manner, promoting the proliferation of epithelial cells. Consequently, this prevents endometrial differentiation and hampers proper embryo implantation (82). Additionally, M2 macrophage exosomes, which contain elevated levels of circRNA-Ep400, suppress the activity of miR-15b-5p. This suppression leads to an upregulation of FGF1/7/9 expression in fibroblasts and tendon cells (83). In aged skin, poly-D, L-lactic acid (PDLLA) triggers the activation of the nuclear factor (erythroid-derived 2)-like 2 factor (Nrf2) signaling pathway in macrophages, leading to M2 polarization and IL-10 expression in senescent macrophages. Consequently, the increased release of IL-10 by macrophages stimulates the growth and secretion of FGF2 and TGF-β in adipose-derived stem cells (ASCs). These growth factors, in turn, may play a role in the polarization and functional control of macrophages (84).

In addition to its role in various inflammatory diseases, FGF signaling is also significantly altered in macrophage-associated inflammatory diseases. Inappropriate interactions between macrophages and T cells are often linked to the pathogenesis of RA, where classical (M1) macrophage activation can impact the development of T-helper (Th)1 responses (85). Single-cell RNA sequencing analyses have shown high activation of the FGF pathway in lining fibroblast-like synoviocytes (FLSs) from patients with relapse RA. Furthermore, multiplex immunohistochemistry (mIHC) has confirmed enhanced expression of FGF10 in these FLSs. Knockdown of FGF10 in FLSs has been found to significantly reduce the expression of NF-κB ligand receptor activator, thereby alleviating collagen-induced arthritis (86). Additionally, macrophages have been identified as key drivers of inflammation in inflammatory bowel disease (IBD), with FGF15 being significantly down-regulated in intestinal tissues of a dextran sodium sulfate (DSS)-induced colitis model (53). While these studies have observed changes in FGF expression in macrophage-related inflammatory diseases, the direct relationship between FGF signaling and macrophage polarization remains to be fully elucidated.

### 4.2 FGFs facilitate macrophage M1 polarization

**Figure 4:**
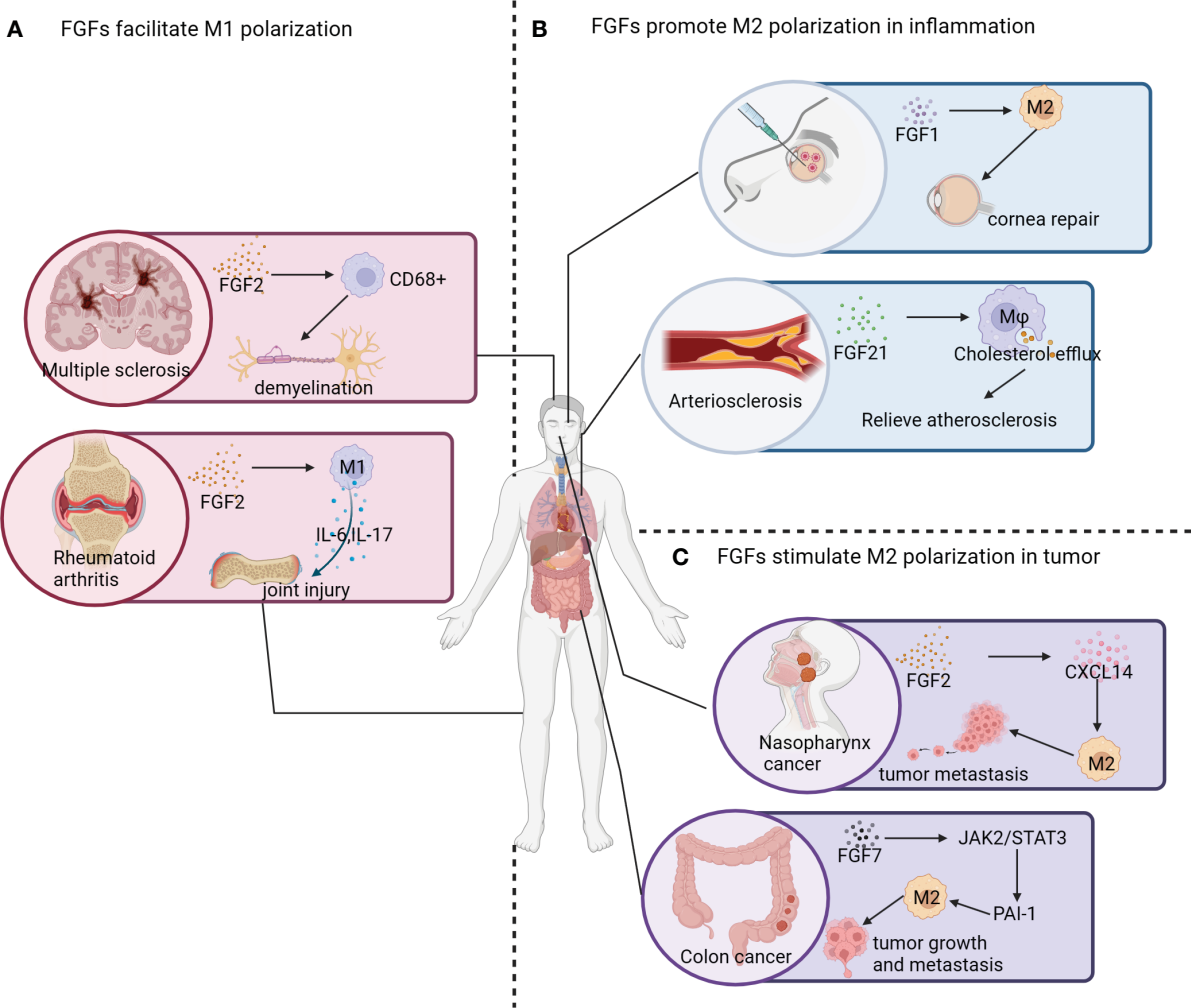
Effect of FGF signaling pathway on macrophage polarization. **(A)** FGF2 contributes to neural demyelination and the development of multiple sclerosis by increasing the number of CD68+ macrophages. In patients with rheumatoid arthritis, increased expression of FGF2 promotes M1 polarization of macrophages and the release of inflammatory factors (*e.g.* IL-6 and IL-17), leading to joint damage. **(B)** The application of engineered FGF1 (TTHX1114) displays significant efficacy in relieving corneal damage caused by corneal herpes; and FGF21 has been found to induce macrophage cholesterol efflux and slow down the progression of atherosclerosis. **(C)** In nasopharyngeal cancer survivors, elevated FGF2 expression in nasopharyngeal tissues leads to the activation of CXCL14, which promotes the polarization of macrophages into the M2 phenotype and facilitates tumor metastasis. FGF7 activates the JAK2/STAT3 pathway to upregulate PAI-1 expression, promoting M2 polarization of macrophages and facilitating tumor growth and metastasis.

The high plasticity of macrophages enables them to perform various functions in response to environmental changes and maintain the internal environment’s homeostasis (11) (**Figure 4A**). In this review, we focus on the effects of FGFs on macrophage polarization and their mechanisms of action in specific environments. FGF2, a globular protein consisting of a single peptide with a molecular weight of 18 kDa, is involved in diverse cellular and metabolic processes, contributing to cellular and metabolic homeostasis (5). In autoimmune diseases like multiple sclerosis (MS), FGF2 plays a crucial role as a modulator (87). Brain tissues of patients with progressive MS showed significantly higher levels of FGF2 compared to normal brain tissues. Within active lesion tissues, FGF1/2-positive macrophages and astrocytes were abundantly clustered both within and around the lesions (88). Moreover, FGFR1 was found to be highly upregulated in oligodendrocyte precursor cells (OPC). During the early stages of MS pathogenesis, FGF2 aids in internal repair, promoting the recruitment of OPCs and myelin production. However, in later stages, FGF2 impedes OPC differentiation (83). In neuroprotective autoimmune processes, FGF2 contributes to inflammation, leading to tissue damage (89). Another study observed that myelin regenerating tissues exhibited high expression of FGF1, while demyelinated tissues showed minimal expression of FGF1, suggesting a potential role of FGF1 in promoting myelin regeneration (90). Additionally, joint tissues affected by RA and collagenous arthritis (CIA) showed significantly elevated levels of FGF2 and IL-17. Furthermore, FGF2 collaborates with IL-17 to accelerate the pathogenesis of RA by inducing an inflammatory response (91).

There is increasing evidence suggesting that FGF23 directly interacts with immune cells such as PMNs and/or macrophages by binding to FGFR/α-Klotho receptors (92). Inflammatory stimuli induce an upregulation of α-Klotho expression in macrophages, which, in turn, helps to restore the FGFR-α-Klotho signaling pathway (13). Treatment with recombinant FGF23 protein results in the induction of TNF-α mRNA and protein expression in macrophages by activating the binary FGFR1c/α-Klotho complex (13).

FGF19 is one of the most frequently amplified genes in HCC patients and has been identified as a potential driver gene of HCC (93). During FGF19-triggered hepatocellular oncogenesis, CD45+ immune cells, and Kupffer cells in particular, are the primary source of IL-6 in response to FGF19 stimulation. Collectively, macrophage-derived IL-6 signaling plays a pivotal role in potentiating FGF19-driven HCC pathogenesis in mice (94).

Several FGFs can be involved in the inflammatory phenotype of macrophages under certain conditions. However, the same FGF may exert different effects on the polarization of macrophages under different contexts.

### 4.3 FGFs stimulate M2 polarization of macrophages in inflammatory diseases

In addition to promoting M2 polarization in TAMs, FGFs also play a role in regulating the direction of macrophage polarization in inflammatory diseases (**Figure 4B**). FGF1, a naturally occurring protein, is crucial for promoting cell proliferation, migration, and cytoprotective properties. These properties are essential for the treatment of corneal diseases as they contribute to the healing process (95). Furthermore, FGF1 seems to convert resident macrophages at the cornea to the M2 phenotype. However, the mechanism underlying this effect is still poorly understood (96).

In murine models of COPD induced by elastase or cigarette smoke exposure, it has been observed that FGF2 has a protective effect on lung function (97). Studies have shown that serum samples from patients with chronic obstructive pulmonary disease have significantly lower levels of FGF2 compared to those from normal subjects. Similarly, mice exposed to short-term cigarette smoke also exhibit reduced expression of FGF2. When FGF2 is administered intranasally, there is a significant reduction in alveolar macrophage and lymphocyte infiltration, suggesting a potential role of FGF2 in mitigating airway inflammation (97). On the other hand, the PD-L1/PD-1 signaling pathway serves as a negative regulatory mechanism in the immune response and plays a crucial role in tumor evasion of immune surveillance and the development of autoimmune diseases (98). Research has found that PD-L1 is widely expressed in fibroblast-like cells present in wound granulation tissue, and inhibiting PD-L1 slows down the healing process. Furthermore, the combination of FGF2 and TGF-β leads to an increase in PD-L1 expression in fibroblast-like cells by activating the PI3k-AKT-mTOR-4EBP1 and p38-ERK-MNK-eIF4E signaling pathways. Consequently, the upregulation of PD-L1 in fibroblast-like cells facilitates the polarization of macrophages from M1 to M2, contributing to the resolution of inflammation and wound healing (99).

Acute lung injury (ALI) is primarily caused by sepsis, resulting in alveolar injury, pulmonary edema, and vascular hyperpermeability, leading to severe hypoxemia (100). Studies have shown that FGF2 effectively reduces the infiltration of macrophages in the lungs of septic mice. Specifically, FGF2 inhibits the expression of inflammatory factors IL-1β, IL-6, and TNF-α by targeting the P38/AKT/NF-κB pathway in LPS-treated macrophages (101). Furthermore, there is a consistent association between depression and inflammation (102). Detailed investigations on postmortem brains of individuals with major depression have revealed decreased expression levels of both FGF2 and FGFR1. Neuroinflammation can suppress the FGF2-ERK1/2 signaling pathway and hinder hippocampal neurogenesis.

FGF4, also known as FGF-K or K-FGF, has demonstrated anti-inflammatory, anti-apoptotic, and glucose- and lipid-lowering effects (103). In adipose tissue, macrophage infiltration and inflammation induced by obesity are critical factors contributing to insulin resistance (104). Recombinant FGF4 (rFGF4) directly affects macrophages by inducing phosphorylation of FGFR1, which subsequently leads to a decrease in the nuclear entry level of IκBα/NF-κB complex and NF-κB subunit P65 (105). Importantly, long-term administration of FGF4 reduces adipose tissue macrophage infiltration and inflammation, resulting in a significant improvement in insulin resistance (105). In the LPS-induced ALI model, treatment with rFGF4 significantly improves the lung W/D weight ratio, survival rate, and reduces lung tissue injury and apoptosis. Subsequent cellular experiments confirm the protective effect of rFGF4 on LPS-induced ALI in mouse lung tissue. This effect is achieved through the inhibition of the TLR4/NF-κB signaling pathway in MH-S (mouse alveolar macrophages) and MLE-12 (mouse lung epithelial cells), as well as the suppression of inflammatory mediators (106). In the pathogenesis of immune-mediated liver injury (ILI), a deficiency in hepatic FGF4 exacerbates apoptosis and leads to an imbalance in intrahepatic immunity, including the accumulation of infiltrated macrophages. Mechanistically, hepatic FGF4 inhibits the infiltration of macrophages and T-cells triggered by apoptosis under ILI conditions (107).

Liver fibrosis is characterized by the activation and proliferation of hepatic stellate cells (HSC), which are the primary source of matrix-producing myofibroblasts (108). In the liver, hepatic macrophages play a crucial role in the differentiation of hepatic stellate cells into hepatic fibroblasts. These macrophages can be categorized into pro-inflammatory, pro-fibrotic, anti-inflammatory, and attenuated fibrotic types (109). Macrophages expressing high levels of Ly6C release several pro-fibrotic cytokines, such as TGF-β, platelet-derived growth factor (PDGF), and chemokine (CC-motif) ligand 2 (CCL2) (110). FGF12, a cytosolic factor belonging to the FGF superfamily, is involved in this process (110). Specifically, FGF12 is up-regulated in hepatic macrophages in mouse models of liver fibrosis induced by bile duct ligation (BDL) and chronic CCL4 injection. Moreover, FGF12 can promote the phenotypic transition of macrophages from low to high Ly6C expression by activating the CCL2/CCR2 axis (110).

FGF18, a high-affinity ligand for FGFR3, plays a pivotal role in organ development and damage repair. FGF18 enhances bone regeneration by modulating the immune response of macrophages and stabilizing bone healing through BMP2. The application of FGF18+BMP2 hydrogel to cranial bone defects promotes the infiltration of M2 macrophages, which are associated with tissue healing before mineralized bone formation and the expression of anti-inflammatory markers. Mechanistically, FGF18 induces M2 polarization of macrophages by stimulating the production of CCL2 during cranial bone healing (111).

FGF21, unlike other members of the FGF family, exhibits weak binding to heparin and is able to cross the blood-brain barrier (112). In a mouse model of L-arginine-induced chronic pancreatitis (CP), treatment with FGF21 significantly improves the inflammatory status of serum, pancreas, and peritoneal macrophages by inhibiting the NF-κB signaling pathway (113). The inflammatory response plays a crucial role in causing secondary damage in ischemic stroke. After a stroke, there is a rapid accumulation and activation of microglia, as well as various immune cells such as monocytes, macrophages, lymphocytes, neutrophils, and NK cells. These cells can cross the blood-brain barrier and release large amounts of inflammatory factors, thereby promoting inflammation (114, 115). Conjugating FGF21 to FGFR1 has been shown to upregulate the expression of peroxisome proliferator-activated receptor (PPAR)-γ and inhibit the NF-κB pathway. This leads to a reduction in the production of IL-1β, TNF-α, IL-6, and COX2, ultimately promoting the conversion of macrophage phenotype and lowering the risk of ischemic stroke (116). In a mouse model of monosodium glutamate (MSG)-induced obesity, FGF21 has been found to have anti-inflammatory effects on various cell types in adipose tissue, including adipocytes, preadipocytes, and macrophages. These effects are mediated by the FRS2/ERK1/2 signaling cascade (117). Additionally, the deposition of macrophages in blood vessels occurs when they absorb low density lipoproteins and form foam cells. This process plays a crucial role in the development of atherosclerosis. Research has shown that the deficiency of FGF21 leads to a significant increase in the formation of atherosclerotic plaques in ApoE-/- mice (118). FGF21 can activate the expression of ABCA1 and ABCG1 in macrophages, which enhances cholesterol efflux from macrophages. This suggests that FGF21 may have a beneficial role in preventing atherosclerosis (119). It is important to note that the effect of FGF21 on macrophage migration and inflammatory response depends on its inhibition of the NF-κB signaling pathway in oxidized low-density lipoprotein (ox-LDL)-induced THP-1 macrophages (120).

In addition, FGFR also plays a role in regulating macrophage polarization. The accumulation of activated macrophages in the synovium and the mediators produced by these macrophages are positively associated with inflammatory and joint destructive responses (121). Studies have shown that the deletion of FGFR3 promotes the recruitment of monocytes/macrophages in the synovial tissues of senescent mice, and this recruitment is closely linked to increased expression of CXCR7. Macrophages deficient in Fgfr3 exhibit an upregulation of CXCR7, which is dependent on the activation of HIF-1α and NF-κB. The FGFR3-CXCR7 pathway in monocytes/macrophages could potentially serve as a new target for arthritis treatment (68). Another study found that treating THP-1-derived macrophages with IS stimulates the production of pro-inflammatory cytokines TNF-α, IL-6, and IL-1β, and induces M1 polarization (122). Furthermore, it was observed that the downregulation of Klotho expression occurs in macrophages treated with IS, and overexpression of Klotho helps alleviate the IS-induced inflammatory response in macrophages by promoting M2 polarization (122).

### 4.4 FGFs promote M2 polarization of TAMs

M2 macrophages play a crucial role in anti-inflammatory responses, tissue remodeling, tumorigenesis, and metastasis (80). Several studies have demonstrated the ability of FGFs to induce macrophage M2 polarization, which subsequently influences tumor microenvironment (TME) and tumor progression (**Figure 4C**).

A variety of cells have the ability to synthesize FGF2, which has been shown to have a significant impact on the proliferation, migration, and differentiation of tissue cells. FGF2 has been found to have strong pro-angiogenic effects both *in vivo* and *in vitro*, promoting the growth of smooth muscle cells and facilitating wound healing and tissue regeneration (123). Several experiments have confirmed that high expression of FGF2 promotes tumor growth, tumor metastasis, and infiltration of M2-type macrophages in a tumor-bearing mouse model and *in vivo* tests (124). FGF2 indirectly promotes the secretion of CXCL14 through the activation of FGFR1/ERK/aryl hydrocarbon receptor (AHR) signaling cascade from pericytes, which in turn induces M2 polarization of TAMs and promotes tumor metastasis (124). Additionally, TAMs, which play a crucial role within the tumor microenvironment, are known to express FGF2. FGF2 exists in various isomers that can be produced from different translation initiation sites. Among these isomers, the lowest molecular weight subtype of FGF2 (FGF2^LMW)^ stands out as the only secreted form of FGF2 identified so far (125). Mice deficient in FGF2^LMW^ experience significant inhibition of tumor metastasis and an increase in the iNOS^+^/CD206^+^ ratio (also known as M1/M2 ratio) in TAM, highlighting that the deletion of FGF2 promotes M1 polarization of macrophages. These findings suggest that FGF2 present in the tumor microenvironment plays a crucial role in regulating macrophage differentiation and may be involved in the reprogramming of TAMs (14).

In the context of esophageal squamous cell carcinoma (ESCC) tissues, TAMs play a crucial role in promoting angiogenesis within the tumor and creating an immunosuppressive microenvironment. Recombinant human FGF2 (rhFGF2) also plays a key role in enhancing the migration and survival of macrophage-like cells (Macrophage-Ls) derived from peripheral blood monocytes and TE cells by activating FGFR1 signaling. The signaling pathway involving NCAM and FGF2-mediated FGFR1 is responsible for regulating the expression of phosphorylated FGFR1 in TAM-Ls (126). Consequently, the upregulation of FGF2 in mesenchymal cells, including macrophages, indicates a more aggressive phenotype and an increased infiltration of M2 macrophages.

TAMs in lung adenocarcinoma are characterized by high levels of FGF2, FGF10, FGFR2, TGF-β, VEGF, and matrix metalloproteinase (MMPs). Additionally, lung adenocarcinoma induced by FGF9 is closely linked to the recruitment and activation of M2-TAMs, which exhibit immune suppressive and proangiogenic functions, thereby facilitating tumor growth (127). Moreover, the FGF7/FGFR2 signaling pathway regulates fibrinogen activator inhibitor-1 (PAI-1), which is associated with a poor prognosis in advanced colorectal cancer (CRC). Mechanistically, FGF7/FGFR2 enhances PAI-1 expression through the JAK2/STAT3 signaling cascade, and the FGFR2/PAI-1 axis promotes M2 polarization of TAMs in the TME of CRC (128).

Ovarian cancer, a highly lethal gynecological tumor, often presents at advanced stages with high recurrence rates. Epithelial ovarian cancer (EOC) accounts for more than 90% of cases. Research has shown that overexpression of FGF18 is an independent predictive marker for poor clinical outcomes in EOC patients. FGF18 signaling accelerates tumor progression by promoting ovarian tumor aggressiveness and altering the TME, leading to increased infiltration and M2 polarization of macrophages. Mechanistically, FGF18 activates FGFR4, triggering NF-κB-mediated cytokine release in ovarian cancer cells, resulting in TAM infiltration and M2 polarization. Additionally, FGF18 expression is closely associated with M2-TAM infiltration in serous ovarian cancer, suggesting its potential as a prognostic and therapeutic biomarker for EOC patients (129).

Human FGF19 and its mouse orthologue FGF15 are gut hormones that exhibit low affinity towards HSPG. These hormones play a crucial role in various biological processes such as the regulation of bile acid homeostasis, carbohydrate metabolism, lipid metabolism, obesity, diabetes, and nephropathy (55, 130). The literature suggests a significant association between FGF19/15 and tumor progression as well as inflammatory diseases, both of which are characterized by macrophage dysregulation. For example, the co-regulation of E-twenty-six-specific sequence variant 4 (ETV4) expression through the ERK1/2 axis has been investigated in the context of FGF19-FGFR4 and HGF/c-MET signaling. ETV4 enhances the expression of PD-L1 and chemokine CCL2 in HCC cells, leading to the infiltration of MDSCs and TAMs, the suppression of CD8+ T cells, and the facilitation of HCC metastasis (70).

FGF20, a paracrine cytokine secreted by glioma cells, has been found to interact with macrophages. Treatment of macrophages with FGF20 leads to a reduction in their pro-inflammatory phenotype when stimulated with LPS and IFN-γ. This reduction is characterized by decreased levels of macrophage M1 markers and a decrease in the production of pro-inflammatory cytokines (131). The mechanism behind this effect involves the interaction between FGF20 and macrophage FGFR1, which activates the GSK3β/β-catenin pathways. It has been observed that glucocorticoid (GC) treatment increases FGF20 expression in glioma cells. Conversely, reducing FGF20 expression in glioma cells significantly impairs the effect of GCs on macrophage polarization. These findings suggest that FGF20, secreted by glioma cells, plays an anti-inflammatory role in glioma treatment by regulating macrophage function mediated by GCs. Additionally, this study reveals a molecular connection between glioma cells and macrophages, indicating that FGF20 modulates GC-induced macrophage dysfunction during glioma development (131).

FGFR3-mutant uroepithelial carcinoma (UC) exhibits a more immunosuppressive tumor microenvironment (TME) with lower immune cell infiltration and T-cell toxicity compared to FGFR3-wildtype UC. Interestingly, higher abundance of TREM2+ macrophages (which tend to polarize towards the M2 phenotype) in FGFR3-mutant UC leads to the destruction and suppression of T cell activation, contributing to the formation of an immunosuppressive TME. Additionally, FGFR3-mutant status can be used as a biomarker to predict the therapeutic response of metastatic UC to immune checkpoint blockade (ICB). Inhibiting FGFR3 may activate the immune microenvironment, and combining FGFR inhibitor with ICB could be a promising therapeutic approach in metastatic UC (132).

In summary, the expression of FGF/FGFRs undergoes significant changes during macrophage polarization, and FGFs regulate macrophage polarization by activating FGFRs, either directly on macrophages or indirectly (**Table 1**). This regulation has implications for organismal homeostasis, wound repair, and tumor progression.

**Table 1:** Effect of FGFs/FGFRs on macrophage polarization.

Disease	Molecular	Signal axis	Effector molecular	Mφ phenotype	Ref.
Herpes cornea	FGF1↓	——	——	M1	(95)
Multiple sclerosis (MS)	FGF2↑	——	CD68+ Mφ↑	M1	(70)
Rheumatoid arthritis (RA)	FGF2↑	ERK1/2 ↑	IL-6, IL-17↑	M1	(91)
Chronic obstructive pulmonary disease (COPD)	FGF2↓	——	——	M1	(97)
Chronic inflammation	FGF2↓	PI3K-AKT-mTOR-4EBP1 /p38-ERK-MNK-eIF4E↓	PD-L1↓	M1	(99)
Acute lung injury	FGF2↓	P38/AKT/NF-kB↑	IL-1β, IL-6, TNF-α↑	M1	(101)
	FGF4↓	TLR4/NF-kB↑	IL-1β, IL-6, TNF-α↑	M1	(106)
Nasopharynx cancer (NPC)	FGF2↑	ERK/AHR↑	CXCL14↑	M2	(124)
Colorectal cancer	FGF7↑	JAK2/STAT3↑	PAI-1↑	M2	(128)
Cranial bone healing	FGF18↑	——	CCL2↑	M2	(111)
Epithelial ovarian cancer (EOC)	FGF18↑	NF-κB↑	TAM↑	M2	(129)
Hepatocellular carcinoma	FGF19↑	ERK1/2↑, ETV4↑	PD-L1, CCL2↑	M2	(70)
Neuroglioma	FGF20↑	GSK3β↑	β-catenin↑	M2	(131)
Obesity	FGF21↑	——	CD68+ Mφ, MCP-1, HIF-1α↑	M1	(118)
Chronic pancreatitis (CP)	FGF21↓	NF-kB↑	IL-1β, IL-6, TNF-α↑	M1	(113)
Cerebral ischemic stroke	FGF21↓	PPARγ↓, NF-kB↑	IL-1β, IL-6, TNF-α, COX2↑	M1	(116)
Atherosclerosis (AS)	FGF21↓	NF-kB↑	ABCA1, ABCG1↓	M2	(119)
Urothelium carcinoma (UC)	FGFR3↑	——	TREM2+ Mφ↑ (M2)	M2	(132)
Arthritis	FGFR3↓	HIF-1α/NF-kB/CXCR7↑	——	M1	(68)
ESCC	FGFR1↑	PI3K/AKT↑	——	M2	(126)

## 5 Potential strategies for regulating macrophage function by manipulating FGF signaling

Currently, the targeted regulation of macrophage polarization and function has emerged as a crucial strategy for treating inflammatory diseases, cancer, metabolic diseases, etc (79). A significant aspect to consider is the pivotal role played by FGF/FGFRs in macrophage polarization. Consequently, the modulation of FGF signaling has become a valuable approach to rectify macrophage dysfunction.

### 5.1 Enhancing FGF signaling

Recombinant FGFs have the ability to mimic the physiological function of FGFs both *in vitro* and *in vivo*. They have shown significant therapeutic potential for various diseases, particularly inflammatory diseases and acute injuries. In a mouse model of LPS-induced ALI, treatment with rFGF4 has been found to effectively inhibit the activation of the TLR4/NF-κB signaling pathway and the production of pro-inflammatory mediators in the injured lung tissues. Additionally, rFGF4 has been shown to regulate the TLR4/NF-κB signaling pathway in murine alveolar macrophages and murine pulmonary epithelial cells, resulting in a decrease in the generation of inflammatory mediators such as IL-1β, IL-6, TNF-α, and COX-2 (106). Moreover, intranasal treatment with rFGF2 has been found to significantly reduce macrophage-dominant inflammation and improve alveolar destruction in a mouse model of COPD. Importantly, no serious adverse events were reported during the treatment of COPD patients with inhaled rFGF2 (97). A novel bFGF (also known as FGF2) containing hydrogel, HA-bFGF, has shown reparative effects *in vivo* on tissue damage following spinal cord injury. This bFGF-loaded hydrogel promotes myelin regeneration in Schwann cells, reduces inflammation at the injury site, and ultimately enhances axon production (133).

Recombinant human (rh) FGF21 shows promise as a drug candidate for treating diabetes, promoting wound healing, and addressing metabolic dysfunction. It has been found that rhFGF21 not only inhibits macrophage-mediated inflammation by activating the Nrf2-mediated anti-oxidant response and suppressing the NF-κB signaling pathway but also reduces the levels of pro-inflammatory cytokines in diabetic mouse and human corneal epithelial cells (134). Additionally, rhFGF21 treatment decreases reactive oxygen species production, boosts anti-inflammatory molecules like IL-10 and SOD-1, and reduces the release of inflammatory mediators and matrix metalloproteinases, indicating its potential protective role in healing diabetic corneal epithelial cells through enhanced antioxidant capacity (135).

Engineered FGF analogues also show great promise in disease therapy. One specific application worth mentioning is the use of engineered FGF1 (TTHX1114) to exert its anti-inflammatory effect. This has shown promise in the treatment of primary and recurrent corneal herpes by reducing the infiltration of pro-inflammatory M1 macrophages at the corneal site (96). Furthermore, to investigate the effects of FGF19-M52, a novel protein-engineered mutant, on colitis, scientists injected AAV-FGF19-M52 or control AAV-GFP into WT and FXR-null mice, respectively, and induced colitis using DSS. The results demonstrated that FGF19-M52 reduced bile acid synthesis, protected mice from DSS-induced intestinal inflammation, decreased the expression of pro-inflammatory genes and immune response in the mucosa, maintained intestinal integrity, promoted the transfer of beneficial microorganisms, and contributed to the resolution of the inflammatory phenotype (136). PsTag-FGF21, a long-acting FGF21 analogue, has shown positive therapeutic effects for metabolic dysfunction-associated steatohepatitis (MASH). Specifically, PsTag-FGF21 has been found to significantly reduce hepatic fibrosis in two MASH-fibrosis models by modulating the NR4A1-mediated Ly6C phenotypic switch in liver macrophages (137).

In addition to recombinant FGFs, gene editing approaches have also been considered to enhance FGF signaling. This can help overcome the drawbacks of short half-life, repeated delivery, and the need for multiple injections of recombinant FGFs. By utilizing chondrocyte affinity peptide (CAP)-conjugated heteroexosomes (CAP/FGF18-hyexo) loaded with FGF18-targeting gene editing tools, a CRISPR/Cas9-based approach efficiently activates the FGF18 gene in osteoarthritis chondrocytes *in vivo*. This activation provides long-lasting lubrication during frictional wear (138).

### 5.2 Blocking FGF signalings

On the other hand, it has been found that blocking FGFs using antibodies or inhibitors can effectively address macrophage dysfunction. Notably, clinical studies have demonstrated that radiotherapy can increase FGF2 levels in patients with rectal and cervical cancers (139, 140). In an experiment involving MC38 xenografts in mice, the use of an anti-FGF2 antibody alone did not impact tumor growth in unirradiated mice. However, when combined with fractionated radiation, the anti-FGF2 antibody treatment significantly delayed tumor growth *in vivo*, resulting in improved long-term survival and a higher ratio of M1-TAMs compared to irradiation alone (14). These findings have underscored the potential of administering a combination of radiotherapy and targeted FGF2 antibodies to enhance patient survival (14).

FGFR inhibitors are a novel class of targeted therapies that show promise in modulating inflammatory responses (12). In LPS-stimulated macrophages, the immunoproteasome is upregulated, leading to an NF-κB-mediated inflammatory cascade characterized by increased production of pro-inflammatory cytokines, reactive oxygen species, and nitrogen oxides. LY2874455, a pan-FGFR inhibitor, activates the autophagy pathway, which sequesters the immunoproteasome, thereby inhibiting NF-κB activity and dampening inflammation (141). Moreover, FGFR1 inhibitors such as PD173074 demonstrate potential in mitigating bone erosion in human-derived cell cultures. Targeting the FGF10/FGFR1 axis may offer new therapeutic avenues for patients with RA (86). Similarly, AZD4547, a selective FGFR1 inhibitor, shows promise in preventing cardiac inflammation, fibrosis, and dysfunction in diabetic mouse models (142). Furthermore, infigratinib, an oral pan-FGFR inhibitor, exhibits efficacy in reducing clinical episodes of experimental autoimmune encephalomyelitis by modulating immune cell infiltration, promoting oligodendrocyte maturation, and enhancing myelin regeneration in the spinal cord (87).

The combination of the infigratinib and the selective FGFR4 inhibitor FGF401 has been shown to convert the immunosuppressive microenvironment into an immune-supportive microenvironment. This conversion enables increased infiltration of CD68+ macrophages, CD4+ and CD8+ T cells, CD11c+ dendritic cells, and CD4+ granzyme B into the tumor. As a result, tumor regression is improved compared to the single agent treatment group. Additionally, the FGFR4 inhibitor BLU-554, when combined with anti-PD-L1, effectively restrains HCC metastasis. This effect is partly attributed to the reduction in TAMs (70).

Thus, recombinant FGFs, gene editing, blocking antibodies, and specific FGFR inhibitors are promise in regulating macrophage function in certain diseases (**Table 2**), by correcting disordered FGF/FGFR signaling.

**Table 2:** Modulation of the FGF/FGFR signaling pathway to regulating macrophage polarization.

Strategies	Type	Mφ phenotype	Disease	Ref.
Recombinant FGF	rFGF2	M2	Chronic obstructive pulmonary disease (COPD)	(97)
	HA-bFGF	M2	Spinal cord injury	(133)
	rFGF4	M2	Acute lung injury (ALI)	(106)
	rFGF21	M2	Diabetic neuropathy	(135)
Engineered FGF analogue	TTHX1114 (FGF1)	M2	Herpes cornea	(96)
	FGF19-M52	M2	Enteritis	(136)
	PsTag-FGF21	M2	metabolic dysfunction-associated steatohepatitis (MASH)	(137)
Gene editing	CAP/FGF18-hyEXO/CRISPR-Cas9	M2	Osteoarthritis	(138)
Blocking Antibody	anti-FGF2	M1	Colorectal cancer	(14)
Inhibitor of FGFRs	AZD4547 (anti-FGFR1)	M2	Diabetic heart disease	(142)
	Infigratinib (pan-FGFR inhibitor) + FGF401 (anti-FGFR4)	M1	——	(70)
	BLU554 (anti-FGFR4) + anti-PD-L1	M1	Hepatocellular carcinoma (HCC)	(70)
	Infigratinib (pan-FGFR inhibitor)	M2	Multiple sclerosis (MS)	(87)
	LY2874455 (pan-FGFR inhibitor)	M2	——	(141)

## 6 Conclusion and perspective

Inappropriate FGF signaling has been implicated in various human diseases, such as breast cancer, chondrodysplasia, gastric cancer, lung cancer, and X-linked hypophosphatemic rickets (1). Moreover, it indirectly contributes to the development of other human diseases by influencing angiogenesis and immune dysregulation, such as RA, MS, and diabetes (10). The immune cells play a crucial role in modulating FGF signaling during inflammation and tissue repair, highlighting its dynamic role in regulating immunity. Extensive research has been conducted to understand the mechanisms underlying the function of the FGF/FGFR signaling pathway in both normal and diseased states of the body.

This review aims to explore the role of FGF/FGFR signaling in influencing macrophage polarization and its impact on disease progression. We also discuss the relationship between the FGF/FGFR signaling axis, macrophage polarization, and the pathogenesis of various human diseases, including dysplastic diseases, metabolic disorders, inflammatory diseases, degenerative diseases, injury and regeneration, as well as different types of cancer. Additionally, the document outlines existing therapies and pre-clinical strategies for diseases linked to macrophage dysfunction through the modulation of FGF signaling.

Certainly, further research is needed to investigate the interaction between macrophages and FGF signaling. Specifically, the following aspects could be explored: 1) The role of FGFs as metabolic regulators and their potential influence on macrophage polarization by modulating body physiology. 2) The relationship between gut microbes and macrophages, which are known to be closely linked and involved in modulating intestinal homeostasis. It is unclear whether FGFs play a role in this interaction. 3) The possibility of FGFs transforming cold tumors into hot tumors by regulating TAMs. 4) The potential of FGFR inhibitors, which have been developed for tumor treatment, to also modulate tumor progression by affecting macrophage polarization. 5) The impact of FGFs on macrophage epigenetic modifications and whether they can alter the direction of macrophage polarization. All of these questions are worth exploring to gain a better understanding of the interplay between FGF signaling and macrophages. The FGF/FGFR signaling pathway plays a crucial role in the polarization of macrophages. Targeting this pathway could provide a powerful tool for therapeutically manipulating diseases that involve dysfunctional macrophages.

## Author contributions

LS: Writing – original draft. YL: Writing – review & editing. HZ: Writing – original draft, Writing – review & editing.
